# Towards the use of an amino acid cleavable linker for solid-phase chemical synthesis of peptides and proteins†

**DOI:** 10.1039/d2ob02198f

**Published:** 2022-12-23

**Authors:** Davide Cardella, Yu-Hsuan Tsai, Louis Y. P. Luk

**Affiliations:** a School of Chemistry, Cardiff University Cardiff CF10 3AT UK lukly@cardiff.ac.uk; b Institute of Molecular Physiology, Shenzhen Bay Laboratory Shenzhen 518132 China tsai.y-h@outlook.com

## Abstract

The synthesis of proteins by solid-phase chemical ligation (SPCL) suffers from the paucity of linkers that can be cleaved under mild conditions. Here, we deployed a spontaneous nickel-assisted cleavage (SNAC) tag, known to undergo spontaneous cleavage in the presence of nickel(ii), as a linker for C-to-N SPCL.

## Abstract 2

The use of NiCl_2_ as a mild cleavage reagent in solid-phase peptide and protein synthesis was investigated. Using this approach, two model peptides and a ubiquitin derivative were successfully synthesised and cleaved from a solid support.

## Graphical abstract

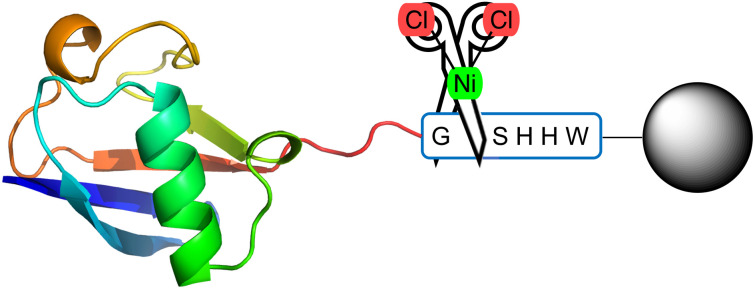

Solid-phase chemical ligation (SPCL) is a powerful method used in protein synthesis.^1,2^ It combines the idea of solid phase chemistry with the advantage of chemical ligation to yield polypeptide segments by elongation on a resin. The clear advantage of SPCL over solution-based chemical ligation is the avoidance of intermediate isolation which is typically low-yielding and time-consuming.^3^ Indeed, shortly after the report of native chemical ligation (NCL), the total synthesis of polypeptides and proteins such as C5a, MIF and vMIP I by SPCL was implemented,^2,4^ whereby unreacted peptide segments, reagents and by-products were removed by simple washing and filtration.

Chemical synthesis in the solid phase requires the use of cleavable linkers to release the product from the solid support. Despite its appealing potential, SPCL is not popularly used, because the choice of a suitable linker is rather limited. Traditionally, cleavage of a polypeptide is achieved by using strong acids, bases, or nucleophiles such as TFA, NaOH, NH_2_OH and hydrazine.^2,3,5^ Recently, cleavage has been achieved by the use of the enzyme phosphatase.^6^ Although the conditions were milder, protein release was found to be slow, taking up to 6 days. Alternatively, a succinimide linker can be used, but it is only suitable for polypeptides carrying cysteine residues.^7^ Recently, a photolabile linker has been reported; however, this approach requires chemical synthesis of a 2-nitrobenzyl derivative.^8^

Here, we have assessed the use of a short amino acid sequence known to undergo spontaneous nickel-assisted cleavage (SNAC)^9^ as a cleavable amino acid linker for chemical synthesis in the solid phase. This sequence was found to undergo spontaneous cleavage at the Gly–Ser junction in aqueous buffer containing NiCl_2_.^9^ Because it is comprised of amino acid residues –GSHHW– only, the SNAC-tag can be either directly added to the resin or inserted into the sequence of the C-terminal peptide segment of interest, without the need to conduct chemical synthesis. Furthermore, the tag does not contain a cysteine residue thereby avoiding an unwanted nucleophilic or disulfide-bond forming reaction. Instead, the product would contain an extra C-terminal glycine residue when released, which unlikely affects the activity of most proteins. The SNAC reaction was recently used for the release of short d-peptides from a streptavidin resin.^10^ In this work, we aimed to investigate the efficiency of the SNAC for the release of constructs of different sizes, ranging from a small peptide to a protein derivative.

To this purpose, a model peptide 1 bearing the SNAC-tag was synthesised *via* SPPS (see the ESI†), converted into peptide-azide,^11,12^ and ligated onto a PEG-based resin (ChemMatrix®) through SPCL (Fig. 1a). The resin was equipped with a Rink amide linker to allow rapid cleavage by TFA treatment and quantification of SNAC. Furthermore, it was functionalised with a trialanine spacer, which distances the SNAC site from the resin enabling access for cleavage reagents. A thiazolidine residue was then added to the resin and converted into a cysteine, following the attachment of the peptide onto the solid support by SPCL. To assess the successful grafting of the model peptide on the resin, an analytical TFA cleavage was performed. LC/MS analysis of the TFA cleavage crude confirmed the desired ligation product 2. The SNAC was then tested using buffer as previously reported,^9^ at either room temperature or 40 °C for 24 h. The release of peptide 3 was monitored by LC/MS and its UV absorbance at 214 nm was recorded after 0, 0.75, 1.5, 3, 6, 12 and 24 hours from the start of the SNAC (Fig. 1b). As a result, the same amount of peptide was released, but the rate of the cleavage performed at 40 °C was higher than the one at room temperature.

**Fig. 1:**
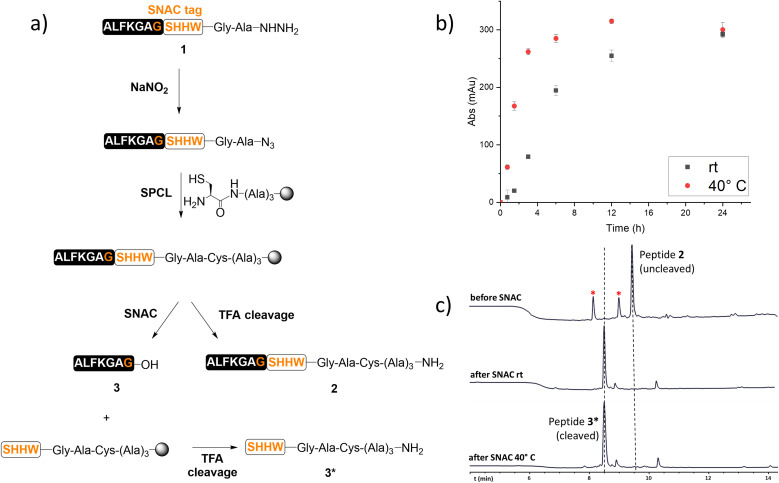
(a) The conversion of model peptide-hydrazide 1 into peptide-azide, and its attachment to a solid support by SPCL, to yield peptide 2. SNAC afforded the desired cleaved peptide 3. (b) Release of the model peptide 3 from the resin *via* SNAC, quantified by integrating the peak area corresponding to peptide 3 from the 214 nm LC chromatogram trace of the cleavage at different time points. (c) LC chromatograms recorded at 280 nm of analytical cleavage using TFA/TIS/DODT/H_2_O (92.5 : 2.5 : 2.5 : 2.5) at room temperature for 2 hours of the model peptide before and after SNAC. *Oxidised product.

After 24 h, a TFA cleavage was performed to check for the cleaved or uncleaved peptide still on the resin after the SNAC. Gratifyingly, while a peak corresponding to the cleaved peptide 3* was found, no uncleaved peptide 2 could be detected (Fig. 1c).

Next, we wanted to test SNAC on a bigger construct 4 (Table 1). For its preparation, peptide blocks 4a–c (Table 1) were synthesised by SPPS (see the ESI†). The Rink Amide ChemMatrix resin was chosen as a water-compatible support and functionalised with a trialanine spacer and a thiazolidine residue, to allow SPCL of the first peptide block 4a to the resin (Fig. 2). After unmasking of the N-terminal cysteine residue of the attached 4a, the peptide segments 4b and 4c were sequentially ligated in the same fashion. Then, SNAC was carried out for 24 h at 40 °C, followed by the addition of TCEP. The reaction resulted in 97% cleavage of the peptide from the resin (ESI Fig. 1†) and afforded peptide 4 in 34% yield (not isolated, calculated *via* integration of the corresponding peak in the LC/MS chromatogram recorded at 214 nm, see the ESI†).

**Fig. 2:**
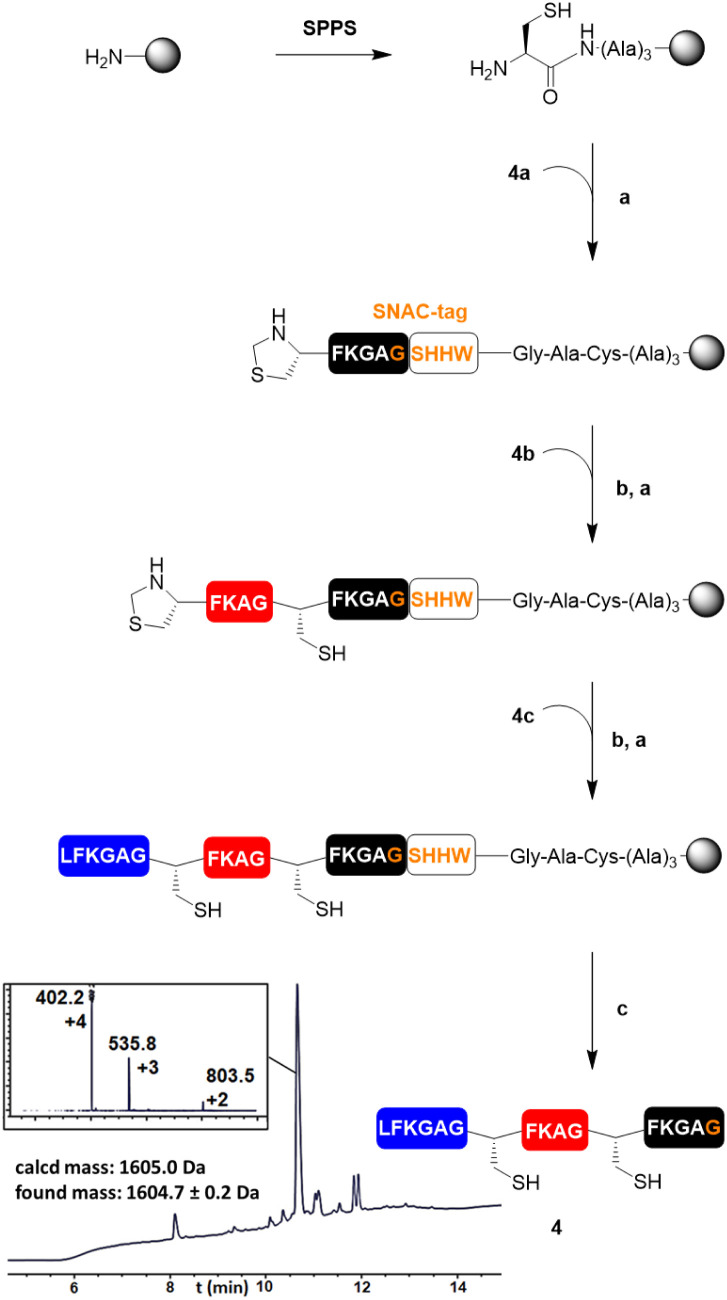
Synthetic scheme for the preparation of model polypeptide 4. The LC/MS trace recorded at 214 nm of the crude cleavage mixture was obtained after incubation of the resin with SNAC buffer for 24 h at 40 °C. (a) 1.5 eq. of thiodepsipeptide, 0.2 M K_2_HPO_4_, 6 M GdnHCl, 0.2 M MPAA, 50 mM TCEP, pH 6.9, 16 h; (b) 0.4 M MeONH_2_, 25 mM TCEP, 40 °C, 16 h; (c) (i) NiCl_2_·6H_2_O (4 eq.), 0.1 M CHES, 0.1 M NaCl, 0.1 M acetone oxyma, pH 8.6, 24 h, 40 °C; (ii) 0.1 M TCEP, 30 min, 40 °C. (See the ESI† for the LC/MS traces recorded at 214 nm of the analytical TFA cleavage of the intermediates and of the crude mixture after SNAC with mass spectra of the side peaks.)

**Table 1:** Sequences of model polypeptide 4, ubiquitin derivative 5 and their peptide segments

Peptide #	Peptide sequence
4	LFKGAGCFKAGCFKGAG-NH_2_
4a	LFKGAGC-MPAA
4b	ThzFKAG-MPAA
4c	ThzFKGAGSHHWGA-MPAA
5	NleQIFVKTLTGKTITLEVEPSDTIENVKCKIQDKEGIPPDQQRLIFCGKQLEDGRTLSDYNIQKESTLHLVLRLRGG-NH_2_
5a	ThzGKQLEDGRTLSDYNIQKESTLHLVLRLRGG̲S̲H̲H̲W̲GA-MeNbz-G-NH_2_
5b	ThzKIQDKEGIPPDQQRLIF-MeNbz-G-NH_2_
5c	NleQIFVKTLTGKTITLEVEPSDTIENVK-MeNbz-G-NH_2_

Towards assessing the use of the SNAC-tag for the cleavage of proteins from a solid support, we sought to synthesise ubiquitin by SPCL. Ubiquitin is an 8.6 kDa protein “ubiquitously” found in eukaryotic cells and is responsible for regulatory tasks in several signalling processes.^13^ Its most well-known function is the regulation of protein degradation: when one or more ubiquitin molecules are enzymatically attached to a protein, the protein is delivered to the proteosome complex, where it is degraded and recycled.^13^ As a small protein whose synthesis is well established, ubiquitin is often used as a model for the development of new synthetic procedures.^7,14–16^

A derivative of ubiquitin 5 was prepared here. Met1 was replaced with norleucine (to avoid oxidative side-reactions at the methionine sulphur). Furthermore, Ala28 and Ala46 were replaced with cysteine residues to provide reaction sites for SPCL. Peptides 5a–c were synthesised by SPPS (see the ESI†) and they served as building blocks for the synthesis of the target protein (Table 1). The Rink Amide PEGA resin was chosen as a water-compatible support and functionalised with a trialanine spacer and a thiazolidine residue, to allow the attachment of the first peptide block 5a to the resin (Fig. 3). Following the unmasking of the N-terminal cysteine residue, blocks 5b and 5c were sequentially ligated in the same fashion. Then, SNAC was carried out for 24 h at 40 °C, followed by the addition of TCEP to afford the ubiquitin derivative 5 in 7% isolated yield.

**Fig. 3:**
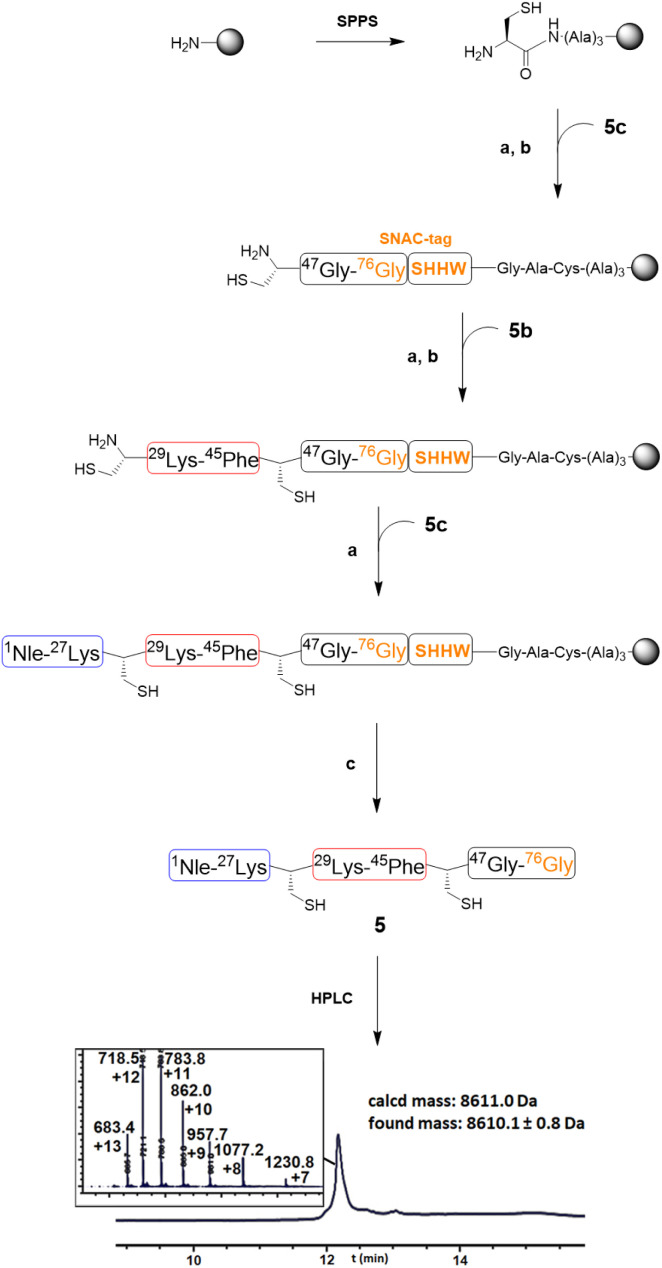
Synthetic scheme for the preparation of the ubiquitin derivative 5. The LC/MS trace for the cleaved product 5 was obtained after incubation of the resin with SNAC buffer for 24 h at 40 °C, followed by HPLC purification. (See the ESI† for the LC/MS traces of the intermediates recorded at 280 nm of the analytical TFA cleavage.) (a) 1.5 eq. of thiodepsipeptide, 0.2 M K_2_HPO_4_, 6 M GdnHCl, 0.2 M MPAA, 50 mM TCEP, pH 6.9, 16 h (the ligation step was performed twice); (b) 0.4 M MeONH_2_, 25 mM TCEP, 40 °C, 16 h; (c) (i) NiCl_2_·6H_2_O (4 eq.), 0.1 M CHES, 0.1 M NaCl, 0.1 M acetone oxyma, pH 8.6, 24 h, 40 °C; (ii) 0.1 M TCEP, 30 min, 40 °C.

The reaction yield is lower than those obtained from existing SPCL protocols,^3,4,6,7^ prohibiting us to examine the possibility of desulfurizing the ubiquitin variants. A low yield is attributed to the partial conversion of the last ligation reaction, as the unreacted intermediate was detected by LC/MS of the analytical cleavage after the ligation reaction (see the ESI, section 3†). The decreased ligation performance, when the size of the immobilised protein or peptide intermediate increases, is in fact a well-known limitation of SPCL syntheses.^2,17–19^ Furthermore, after the SNAC, cleavage was not complete, as the uncleaved protein could be detected by LC/MS following an analytical TFA cleavage (ESI Fig. 2†). Interestingly, along with the uncleaved protein, product 5 was also found (ESI Fig. 2†). The presence of the ubiquitin derivative 5 in the TFA cleavage might be due to the interaction between the PEG-based resin and the target compound. After the SNAC, the protein could be extracted by the organic acid, rather than being cleaved. The protein–resin interaction broken by TFA after the total synthesis of a protein by SPCL is in fact already reported in the literature.^17^

SPCL was reported with the aim of overcoming the need to purify the intermediate during chemical protein synthesis.^2^ Thus far, most of the cleavable linkers have required harsh conditions for the release of the product from the solid support,^2,3,5,20,21^ and this hampers the preparation of constructs bearing labile chemical motifs, or of proteins in their native conformations. Here, we explored the suitability of the SNAC-tag^9^ as a linker that can be cleaved under relatively mild conditions, without the need for chemical synthesis for its preparation and a cysteine residue in the target sequence. Constructs of different lengths were synthesised on PEG-based resins, equipped with a Rink Amide linker. In all cases, the SNAC buffer led to the cleavage of the desired sequence from the tag-functionalised resin. However, incomplete cleavage of the ubiquitin derivative still leaves room for the optimisation of the SNAC. The results suggest that the cleavage of bigger constructs, such as compound 5, might need more SNAC iterations or adjustment of the number of equivalents of NiCl_2_. Furthermore, particular attention will be needed to improve the release of the protein of interest from the resin, in the case of interaction with the solid support. If non-specific binding interactions are thought to occur between the desired product and the resin, repeated washing with TFA (if tolerated by the target protein), heating, or washing with cleavage buffer added with small amounts of tensioactive or chaotropic agents might be considered. Finally, the tolerance of SNAC towards C-terminal amino acid residues should be explored in constructs that range from small peptides to bigger proteins. Although we regard the addition of a C-terminal glycine to a protein sequence as a reasonable modification, a thorough scope will allow accessing constructs with different C-terminal residues. We envision that the described SNAC technology will be complementary to the existing cleavage techniques offering facile access to synthetic proteins.

## Author contributions

D. C. performed the experimental work. Conceptualisation of the project was done by L. Y. P. L. and D. C. Supervision and funding acquisition were carried out by L. Y. P. L and Y. H. T. Writing – original draft was done by D. C. Writing – review and editing was carried out by L. Y. P. L and Y. H. T.

## Conflicts of interest

There are no conflicts to declare.

## Supplementary Material

OB-021-D2OB02198F-s001
