# Supplementary material for: Towards the use of an amino acid cleavable linker for solid-phase chemical synthesis of peptides and proteins
Source: Org Biomol Chem. 2022 Dec 23;21(5):966–9. doi: 10.1039/d2ob02198f (PMC9890637; doi:10.1039/d2ob02198f)
Supplement: OB-021-D2OB02198F-s001 [file OB-021-D2OB02198F-s001.pdf]

Electronic Supplementary Material (ESI) for Organic & Biomolecular Chemistry.
This journal is © The Royal Society of Chemistry 2022

Table of Contents

1. Experimental Section 2

1.1 SPPS MW cycle and method descriptions of CEM Liberty Blue™ Automated Microwave Peptide Synthesizer 2

1.2 SPPS procedure for peptide 1 9

1.3 SPPS procedure for polypeptide H-LFKAGCFKAGCFKGAG-NH_2 for calibration curve 10

1.4 SPPS procedure for peptide 4a 11

1.5 SPPS procedure for peptide 4b 12

1.6 SPPS procedure for peptide 4c 13

1.7 SPPS procedure for peptide 5a 14

1.8 SPPS procedure for peptide 5b 15

1.9 SPPS procedure for peptide 5c 16

2. LC/MS chromatograms for the synthesis of compounds **4** 18

3. LC/MS chromatograms for the synthesis of compounds **5** 22

4. Supplementary figures 24

5. References 25

1. Experimental Section

General Methods. Solid Phase Peptide Syntheses were performed on a CEM Liberty Blue™ Automated Microwave Peptide Synthesizer and in TELOS Filtration Columns equipped with 20 µm polyethylene frits. Reaction temperatures are stated as heating device temperature of the synthesizer, if not otherwise stated. Deionized water was obtained by an Elga PURELAB 8 Option system (15 MΩ·cm). Reagents obtained from commercial suppliers were used without further purification unless otherwise stated. Protected amino acids, Rink Amide MBHA resin, DIC, oxyma pure, piperazine, trifluoroacetic acid and triisopropylsilane (TIS) were purchased from Fluorochem. Fmoc-MeDbz-OH was purchased from Iris Biotech GmbH. DMF was purchased from Fisher Scientific. Fmoc-Asp(OtBu)-(Dmb)Gly-OH, Rink Amide ProTide (LL) and Cl-TCP(Cl) ProTide resins were purchased from CEM Corporation. For HPLC mobile phase, HPLC grade CH_3CN from Fisher Scientific and trifluoroacetic acid from Fluorochem were used. For LC/MS mobile phase, LC/MS grade water and CH_3CN from Fisher Scientific and formic acid from Fisher Scientific were used. Reagents were used without further purification unless otherwise noted. LC/MS was performed on an Agilent 1260 Infinity II HPLC system, equipped with an ACE 3 C18, 2.1 x 100 mm, particle size 3 µm, pore size 300 Å and connected to a 6120 Quadrupole MS. Preparative HPLC was performed on a LC20-AR Shimadzu HPLC system equipped with either a Vydac 208TP C8, 22 x 250 mm, particle size 10 µm, pore size 300 Å or a Shim-pack GIST C18, 20 x 150 mm, particle size 5 µm, pore size 100 Å and semi-preparative HPLC was performed on an Agilent 1206 Infinity HPLC system equipped with either a Zobrax-300SB C8, 4.6 x 150 mm, particle size 5 µm, pore size 300 Å or with an ACE C18, 10.0 x 250 mm, particle size 5 µm, pore size 100 Å. Determinations of PEGA, and ChemMatrix resins loading and ubiquitin derivative yield were conducted using a ThermoFisher NanoDrop ND-ONE-W spectrophotometer. Absorbance values were measured in 10 mm path-length cuvettes (Fisher Scientific, #11847832).

1.1 SPPS MW cycle and method descriptions of CEM Liberty Blue™ Automated Microwave Peptide Synthesizer

MW cycle 1

Temperature (°C)	Power (W)	Hold time (s)
**25**	0	300
**50**	35	600

MW cycle 2

Temperature (°C)	Power (W)	Hold time (s)
**75**	30	300

MW cycle 3

Temperature (°C)	Power (W)	Hold time (s)
**25**	0	5
**80**	115	20
**86**	70	10
**90**	30	120

MW cycle 4

Temperature (°C)	Power (W)	Hold time (s)
**25**	0	5
**80**	115	20
**86**	70	10
**90**	30	480

MW cycle 5

Temperature (°C)	Power (W)	Hold time (s)
**25**	0	120
**50**	35	600

MW cycle 6

Temperature (°C)	Power (W)	Hold time (s)
**75**	30	420

MW cycle 7

Temperature (°C)	Power (W)	Hold time (s)
**50**	30	30

MW cycle 8

Temperature (°C)	Power (W)	Hold time (s)
**50**	30	180

MW cycle 9

Temperature (°C)	Power (W)	Hold time (s)
**25**	0	300

MW cycle 10

Temperature (°C)	Power (W)	Hold time (s)
**25**	0	600

MW cycle 11

Temperature (°C)	Power (W)	Hold time (s)
**20**	0	5
**78**	100	20
**88**	60	10

MW cycle 12

Temperature (°C)	Power (W)	Hold time (s)
**25**	0	5
**78**	80	20
**88**	50	10
**90**	25	60

MW cycle 13

Temperature (°C)	Power (W)	Hold time (s)
**50**	60	270

Method 1

Step	Operation	Parameters	MW cycle
**1**	Wash	DMF volume: 4 mL	--
**2**	Deprotection (x2)	Deprotection cocktail volume: 2 mL	9+10
**3**	Wash (x4)	DMF volume: 4 mL	--
**4**	Coupling (x2)	AA volume: 1.5 mL, DIC volume: 1 mL, HOBt volume: 0.5 mL	5
**5**	Wash through synthesiser manifold	DMF volume: 4 mL	--

Method 2

Step	Operation	Parameters	MW cycle
**1**	Wash	DMF volume: 4 mL	--
**2**	Deprotection (x2)	Deprotection cocktail volume: 2 mL	9+10
**3**	Wash (x4)	DMF volume: 4 mL	--
**4**	Coupling	AA volume: 1.5 mL, DIC volume: 2 mL, HOBt volume: 1 mL	5
**5**	Wash through synthesiser manifold	DMF volume: 4 mL	--

Method 3

Step	Operation	Parameters	MW cycle
**1**	Wash	DMF volume: 4 mL	--
**2**	Deprotection (x2)	Deprotection cocktail volume: 2 mL	7+8
**3**	Wash (x4)	DMF volume: 4 mL	--
**4**	Coupling	AA volume: 1.5 mL, DIC volume: 2 mL, HOBt volume: 1 mL	5
**5**	Wash through synthesiser manifold	DMF volume: 4 mL	--

Method 4

Step	Operation	Parameters	MW cycle
**1**	Wash	DMF volume: 4 mL	--
**2**	Deprotection (x2)	Deprotection cocktail volume: 2 mL	7+8
**3**	Wash (x4)	DMF volume: 4 mL	--
**4**	Coupling (x2)	AA volume: 1.5 mL, DIC volume: 1 mL, HOBt volume: 0.5 mL	5
**5**	Wash through synthesiser manifold	DMF volume: 4 mL	--

Method 5

Step	Operation	Parameters	MW

			cycle
**1**	Wash	DMF volume: 4 mL	--
**2**	Coupling (x2)	AA volume: 1.5 mL, DIC volume: 1 mL, HOBt volume: 0.5 mL	5
**3**	Wash through synthesiser manifold	DMF volume: 4 mL	--

Method 6

Step	Operation	Parameters	MW cycle
**1**	Deprotection	Deprotection cocktail volume: 2 mL	12
**3**	Wash (x4)	DMF volume: 4 mL	
**4**	Coupling	AA volume: 2.5 mL, DIC volume: 2 mL, Oxyma pure volume: 0.5 mL	3
**5**	Wash through synthesiser manifold	DMF volume: 4 mL	

Method 7

Step	Operation	Parameters	MW cycle
**1**	Deprotection	Deprotection cocktail volume: 2 mL	12
**3**	Wash (x4)	DMF volume: 4 mL	
**4**	Coupling	AA volume: 2.5 mL, DIC volume: 2 mL, Oxyma pure volume: 0.5 mL	1
**5**	Wash through synthesiser manifold	DMF volume: 4 mL	

Method 8

Step	Operation	Parameters	MW cycle
**1**	Wash	DMF volume: 4 mL	--
**2**	Deprotection (x2)	Deprotection cocktail volume: 2 mL	9+10
**3**	Wash (x4)	DMF volume: 4 mL	--
**4**	Coupling (x2)	AA volume: 0.75 mL, HATU volume: 0.5 mL, DIPEA volume: 0.25 mL	6
**5**	Wash through synthesiser manifold	DMF volume: 4 mL	--

Method 9

Step	Operation	Parameters	MW cycle
**1**	Wash	DMF volume: 4 mL	--
**2**	Deprotection (x2)	Deprotection cocktail volume: 2 mL	9+10
**3**	Wash (x4)	DMF volume: 4 mL	--
**4**	Coupling (x3)	AA volume: 0.75 mL, HATU volume: 0.5 mL, DIPEA volume: 0.25 mL	6
**5**	Wash through synthesiser	DMF volume: 4 mL	--

	manifold		

Method 10

Step	Operation	Parameters	MW cycle
**1**	Wash	DMF volume: 4 mL	--
**2**	Deprotection (x2)	Deprotection cocktail volume: 2 mL	9+10
**3**	Wash (x4)	DMF volume: 4 mL	--
**4**	Coupling (x3)	AA volume: 1.5 mL, HATU volume: 1 mL, DIPEA volume: 0.5 mL	6
**5**	Wash through synthesiser manifold	DMF volume: 4 mL	--

Method 11

Step	Operation	Parameters	MW cycle
**1**	Wash	DMF volume: 4 mL	--
**2**	Deprotection (x2)	Deprotection cocktail volume: 2 mL	9+10
**3**	Wash (x4)	DMF volume: 4 mL	--
**4**	Coupling	AA volume: 1.5 mL, HATU volume: 1 mL, DIPEA volume: 0.5 mL	6
**5**	Wash through synthesiser manifold	DMF volume: 4 mL	--

Method 12

Step	Operation	Parameters	MW cycle
**1**	Wash	DMF volume: 4 mL	--
**2**	Deprotection (x2)	Deprotection cocktail volume: 2 mL	11
**3**	Wash (x4)	DMF volume: 4 mL	--
**4**	Coupling (x2)	AA volume: 0.75 mL, HATU volume: 0.5 mL, DIPEA volume: 0.25 mL	6
**5**	Wash through synthesiser manifold	DMF volume: 4 mL	--

Method 13

Step	Operation	Parameters	MW cycle
**1**	Wash	DMF volume: 4 mL	--
**2**	Deprotection (x2)	Deprotection cocktail volume: 2 mL	11
**3**	Wash (x4)	DMF volume: 4 mL	--
**4**	Coupling	AA volume: 1.5 mL, HATU volume: 1 mL, DIPEA volume: 0.5 mL	6
**5**	Wash through synthesiser manifold	DMF volume: 4 mL	--

Method 14

Step	Operation	Parameters	MW cycle
**1**	Deprotection (x2)	Deprotection cocktail volume: 2 mL	11
**2**	Wash (x4)	DMF volume: 4 mL	--
**3**	Coupling	AA volume: 1 mL, HATU volume: 0.2 mL, DIPEA volume: 0.25 mL	4
**4**	Wash through synthesiser manifold	DMF volume: 4 mL	--

Method 15

Step	Operation	Parameters	MW cycle
**1**	Deprotection (x2)	Deprotection cocktail volume: 2 mL	9+10
**2**	Wash (x4)	DMF volume: 4 mL	--
**3**	Coupling (x3)	AA volume: 1 mL, HATU volume: 0.15 mL, DIPEA volume: 0.25 mL, DMF volume: 2 mL	6
**4**	Wash through synthesiser manifold	DMF volume: 4 mL	--

Method 16

Step	Operation	Parameters	MW cycle
**1**	Wash	DMF volume: 4 mL	--
**2**	Deprotection (x2)	Deprotection cocktail volume: 2 mL	7+8
**3**	Wash (x4)	DMF volume: 4 mL	--
**4**	Coupling (x2)	AA volume: 0.75 mL, HATU volume: 0.5 mL, DIPEA volume: 0.25 mL	5
**5**	Wash through synthesiser manifold	DMF volume: 4 mL	--

Method 17

Step	Operation	Parameters	MW cycle
**1**	Wash	DMF volume: 4 mL	--
**2**	Deprotection (x2)	Deprotection cocktail volume: 2 mL	7+8
**3**	Wash (x4)	DMF volume: 4 mL	--
**4**	Coupling	AA volume: 0.75 mL, HATU volume: 0.5 mL, DIPEA volume: 0.25 mL	5
**5**	Wash through synthesiser manifold	DMF volume: 4 mL	--

Method 18

Step	Operation	Parameters	MW cycle
**1**	Wash	DMF volume: 4 mL	--

**2**	Deprotection (x2)	Deprotection cocktail volume: 2 mL	9+10
**3**	Wash (x4)	DMF volume: 4 mL	--
**4**	Coupling	AA volume: 0.75 mL, HATU volume: 0.5 mL, DIPEA volume: 0.25 mL	5
**5**	Wash through synthesiser manifold	DMF volume: 4 mL	--

Method 19

Step	Operation	Parameters	MW cycle
**1**	Wash	DMF volume: 4 mL	--
**2**	Deprotection (x2)	Deprotection cocktail volume: 2 mL	9+10
**3**	Wash (x4)	DMF volume: 4 mL	--
**4**	Coupling (x2)	AA volume: 0.75 mL, HATU volume: 0.5 mL, DIPEA volume: 0.25 mL	5
**5**	Wash through synthesiser manifold	DMF volume: 4 mL	--

Method 20

Step	Operation	Parameters	MW cycle
**1**	Wash	DMF volume: 4 mL	--
**2**	Deprotection (x2)	Deprotection cocktail volume: 2 mL	12
**3**	Wash (x4)	DMF volume: 4 mL	--
**4**	Coupling (x2)	AA volume: 0.75 mL, HATU volume: 0.5 mL, DIPEA volume: 0.25 mL	5
**5**	Wash through synthesiser manifold	DMF volume: 4 mL	--

Method 21

Step	Operation	Parameters	MW cycle
**1**	Wash	DMF volume: 4 mL	--
**2**	Deprotection (x2)	Deprotection cocktail volume: 2 mL	11
**3**	Wash (x4)	DMF volume: 4 mL	--
**4**	Coupling	AA volume: 0.75 mL, HATU volume: 0.5 mL, DIPEA volume: 0.25 mL	5
**5**	Wash through synthesiser manifold	DMF volume: 4 mL	--

Method 22

Step	Operation	Parameters	MW cycle
**1**	Wash	DMF volume: 4 mL	--
**2**	Deprotection (x2)	Deprotection cocktail volume: 2 mL	11
**3**	Wash (x4)	DMF volume: 4 mL	--
**4**	Coupling (x2)	AA volume: 0.75 mL, DIC volume: 0.5 mL, HOBt	3

		volume: 0.25 mL	
**5**	Wash through synthesiser manifold	DMF volume: 4 mL	--

Method 23

Step	Operation	Parameters	MW cycle
**1**	Wash	DMF volume: 4 mL	--
**2**	Deprotection (x2)	Deprotection cocktail volume: 2 mL	7+8
**3**	Wash (x4)	DMF volume: 4 mL	--
**4**	Coupling	AA volume: 1.5 mL, DIC volume: 1 mL, HOBt volume: 0.5 mL	3
**5**	Wash through synthesiser manifold	DMF volume: 4 mL	--

Method 24

Step	Operation	Parameters	MW cycle
**1**	Wash	DMF volume: 4 mL	--
**2**	Deprotection (x2)	Deprotection cocktail volume: 2 mL	7+8
**3**	Wash (x4)	DMF volume: 4 mL	--
**4**	Coupling (x2)	AA volume: 0.75 mL, DIC volume: 0.5 mL, HOBt volume: 0.25 mL	3
**5**	Wash through synthesiser manifold	DMF volume: 4 mL	--

1.2 SPPS procedure for peptide 1

Synthesis scale: 0.1 mmol. Resin: Cl-TCP(Cl) ProTide, loading 0.4 mmol/g – 250 mg. DIC concentration in DMF: 1 M. HOBt concentration in DMF: 1 M. Deprotection cocktail: 20% (v/v) piperidine in DMF + 1% (v/v) formic acid. Fmoc-amino acids in DMF: 0.33 M. Prior to the start of the automated synthesis, the resin is swollen in DMF for 5 min at rt and functionalized with hydrazine hydrate following the previously reported procedure.[1]

Reagent	Method	Reagent concentration during coupling step (mM)
Fmoc-Ala-OH	5	167
Fmoc-Gly-OH	4	167
Fmoc-Trp(Boc)-OH	4	167
Fmoc-His(Trt)-OH	3	220
Fmoc-His(Trt)-OH	2	220
Fmoc-Ser(tBu)-OH	1	167
Fmoc-Gly-OH	1	167
Fmoc-Ala-OH	1	167
Fmoc-Gly-OH	1	167
Fmoc-Lys(Boc)-OH	1	167
Fmoc-Phe-OH	1	167
Fmoc-Leu-OH	1	167
Fmoc-Ala-OH	1	167

The last coupling is followed by a deprotection step, performed using 4 mL of deprotection cocktail with MW cycle 14. Cleavage was done with 10 mL of TFA/water/TIS (95:2.5:2.5) for 2 h. The resin was removed by filtration and the filtrate was concentrated by N_2 flow. The crude product was precipitated with cold diethyl ether, centrifuged (4,000 g for 10 min) and the supernatant was discarded. The precipitate was dissolved in water + 0.1% TFA and purified by HPLC. Product was characterized at the LC/MS.

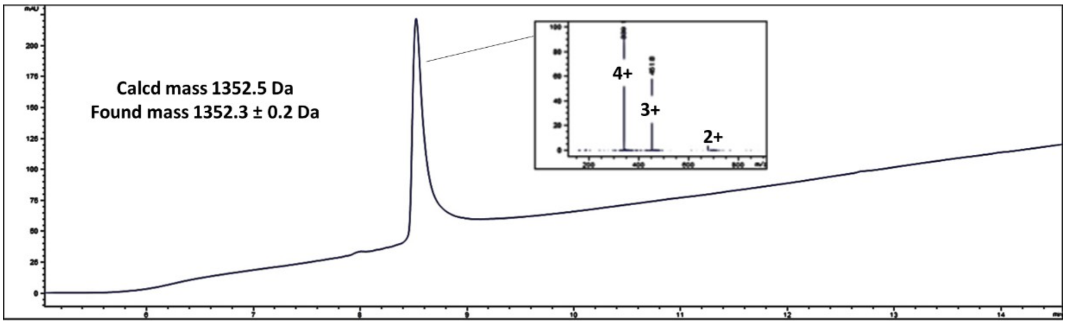

LC chromatogram recorded at 214 nm (+ mass spectrum of the indicated peak) of peptide **1**. Column: *ACE 3 C18*, 2.1 x 100 mm, 3 µm particle size, 300 Å pore size; Method: flow rate = 0.5 mL · min^{-1}, H_2O: CH_3CN, 0.1% HCOOH, 95:5 for 2 min → 95:5 to 43:57 over 13 min → 5:95 for 7 min.

1.3 SPPS procedure for polypeptide H-LFKAGCFKAGCFKGAG-NH_2 for calibration curve

Synthesis scale: 0.1 mmol. Resin: Rink Amide MBHA, loading 0.49 mmol/g – 204 mg. DIC concentration in DMF: 0.5 M. Oxyma pure concentration in DMF: 1 M. Deprotection cocktail: 20% (v/v) piperidine in DMF. Fmoc-amino acids in DMF: 0.2 M. Prior to the start of the synthesis, the resin is swollen in the reaction vessel of the automated synthesizer using 5 mL of DMF for 5 min at rt.

**Reagent**	**Method**	**Reagent concentration during coupling step (mM)**
Fmoc-Gly-OH	6	100
Fmoc-Ala-OH	6	100
Fmoc-Gly-OH	6	100
Fmoc-Lys(Boc)-OH	6	100
Fmoc-Phe-OH	6	100
Fmoc-Cys(Trt)-OH	7	100
Fmoc-Gly-OH	6	100
Fmoc-Ala-OH	6	100
Fmoc-Lys(Boc)-OH	6	100
Fmoc-Phe-OH	6	100
Fmoc-Cys(Trt)-OH	7	100
Fmoc-Gly-OH	6	100
Fmoc-Ala-OH	6	100
Fmoc-Lys(Boc)-OH	6	100
Fmoc-Phe-OH	6	100
Fmoc-Leu-OH	6	100

The last coupling is followed by a deprotection step, performed using 4 mL of deprotection cocktail with MW cycle 14. Cleavage was done with 10 mL of TFA/water/TIS/DODT (92.2:2.5:5:2.5:2.5) for 2 h. The resin was removed by filtration and the filtrate was concentrated by N_2 flow. The crude product was precipitated with cold diethyl ether, centrifuged (4,000 g for 10 min) and the supernatant was discarded. The precipitate was dissolved in water + 0.1% TFA and purified by HPLC. Product was characterized at the LC/MS. See Supplementary Figure 3 for calibration curve.

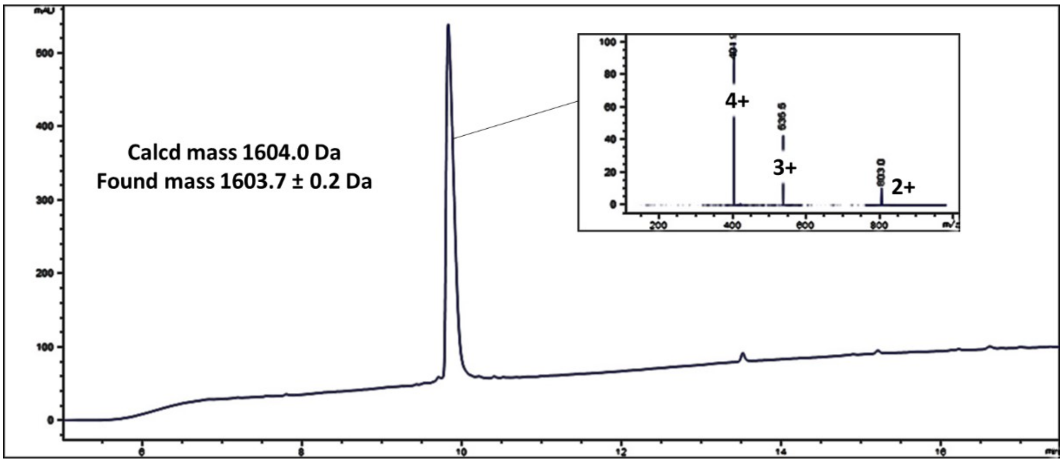

LC chromatogram recorded at 214 nm (+ mass spectrum of the indicated peak) of polypeptide **H-LFKAGCFKAGCFKGAG-NH$_2$**. Column: *ACE 3 C18*, 2.1 x 100 mm, 3 µm particle size, 300 Å pore size; Method: flow rate = 0.5 mL · min^{-1}, H_2O: CH_3CN, 0.1% HCOOH, 95:5 for 2 min → 95:5 to 43:57 over 13 min → 5:95 for 7 min.

1.4 SPPS procedure for peptide 4a

Synthesis scale: 0.1 mmol. Resin: Cl-TCP(Cl) ProTide, loading 0.4 mmol/g – 250 mg. DIC concentration in DMF: 1 M. HOBt concentration in DMF: 1 M. Deprotection cocktail: 20% (v/v) piperidine in DMF + 1% (v/v) formic acid. Fmoc-amino acids in DMF: 0.33 M. Prior to the start of the automated synthesis, the resin is swollen in DMF for 5 min at rt and functionalized with hydrazine hydrate following the previously reported procedure.[1]

Reagent	Method	Reagent concentration during coupling step (mM)
Fmoc-Ala-OH	5	167
Fmoc-Gly-OH	4	167
Fmoc-Trp(Boc)-OH	4	167
Fmoc-His(Trt)-OH	3	220
Fmoc-His(Trt)-OH	2	220
Fmoc-Ser(tBu)-OH	1	167
Fmoc-Gly-OH	1	167
Fmoc-Ala-OH	1	167
Fmoc-Gly-OH	1	167
Fmoc-Lys(Boc)-OH	1	167
Fmoc-Phe-OH	1	167
Fmoc-Leu-OH	1	167
Fmoc-Thz-OH	1	167

The last coupling is followed by a deprotection step, performed using 4 mL of deprotection cocktail with MW cycle 14. Cleavage was done with 10 mL of TFA/water/TIS (95:2.5:2.5) for 2 h. The resin was removed by filtration and the filtrate was concentrated by N_2 flow. The crude product was precipitated with cold diethyl ether, centrifuged (4,000 g for 10 min) and the supernatant was discarded. The precipitate was dissolved in 0.2 M phosphate buffer, containing 6 M GdmCl and the crude product was converted into the corresponding MPAA thioester as previously described.[2] The desired product was then isolated by HPLC. Product was characterized at the LC/MS.

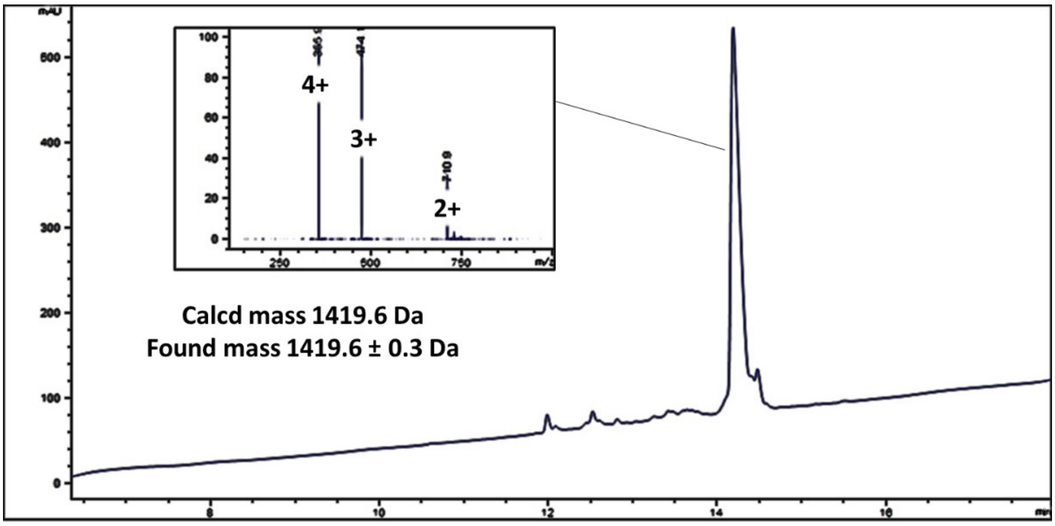

LC chromatogram recorded at 214 nm (+ mass spectrum of the indicated peak) of peptide **4a**. Column: *ACE 3 C18*, 2.1 x 100 mm, 3 µm particle size, 300 Å pore size; Method: flow rate = 0.5 mL · min^{-1}, H_2O: CH_3CN, 0.1% HCOOH, 95:5 for 2 min → 95:5 to 43:57 over 13 min → 5:95 for 7 min.

1.5 SPPS procedure for peptide 4b

Synthesis scale: 0.1 mmol. Resin: Cl-TCP(Cl) ProTide, loading 0.4 mmol/g – 250 mg. DIC concentration in DMF: 1 M. HOBt concentration in DMF: 1 M. Deprotection cocktail: 20% (v/v) piperidine in DMF + 1% (v/v) formic acid. Fmoc-amino acids in DMF: 0.33 M. Prior to the start of the automated synthesis, the resin is swollen in DMF for 5 min at rt and functionalized with hydrazine hydrate following the previously reported procedure.[1]

**Reagent**	**Method**	**Reagent concentration during coupling step (mM)**
Fmoc-Gly-OH	6	100
Fmoc-Ala-OH	6	100
Fmoc-Lys(Boc)-OH	6	100
Fmoc-Phe-OH	6	100
Fmoc-Thz-OH	7	100

The last coupling is followed by a deprotection step, performed using 4 mL of deprotection cocktail with MW cycle 14. Cleavage was done with 10 mL of TFA/water/TIS (95:2.5:2.5) for 2 h. The resin was removed by filtration and the filtrate was concentrated by N_2 flow. The crude product was precipitated with cold diethyl ether, centrifuged (4,000 g for 10 min) and the supernatant was discarded. The precipitate was dissolved in 0.2 M phosphate buffer, containing 6 M GdmCl and the crude product was converted into the corresponding thiophenol thioester as previously described.[2] The desired product was then isolated by HPLC. Product was characterized at the LC/MS.

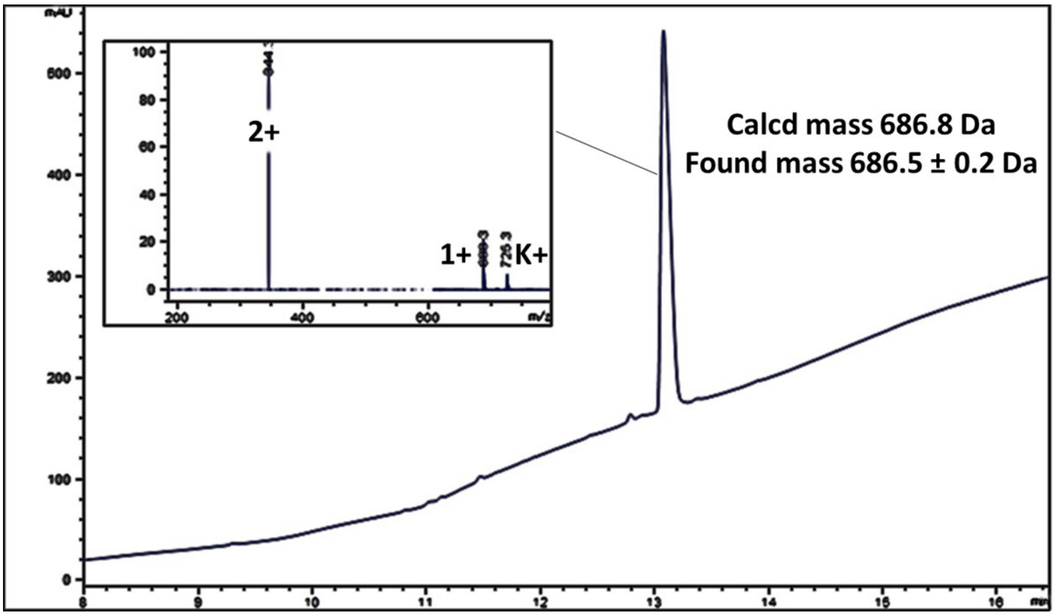

LC chromatogram recorded at 214 nm (+ mass spectrum of the indicated peak) of peptide **4b**. Column: *ACE 3 C18*, 2.1 x 100 mm, 3 µm particle size, 300 Å pore size; Method: flow rate = 0.5 mL · min^{-1}, H_2O: CH_3CN, 0.1% HCOOH, 95:5 for 2 min → 95:5 to 43:57 over 13 min → 5:95 for 7 min.

1.6 SPPS procedure for peptide 4c

Synthesis scale: 0.1 mmol. Resin: Cl-TCP(Cl) ProTide, loading 0.4 mmol/g – 250 mg. DIC concentration in DMF: 1 M. HOBt concentration in DMF: 1 M. Deprotection cocktail: 20% (v/v) piperidine in DMF + 1% (v/v) formic acid. Fmoc-amino acids in DMF: 0.33 M. Prior to the start of the automated synthesis, the resin is swollen in DMF for 5 min at rt and functionalized with hydrazine hydrate following the previously reported procedure.[1]

**Reagent**	**Method**	**Reagent concentration during coupling step (mM)**
Fmoc-Gly-OH	6	100
Fmoc-Ala-OH	6	100
Fmoc-Lys(Boc)-OH	6	100
Fmoc-Phe-OH	6	100
Fmoc-Leu-OH	6	100

The last coupling is followed by a deprotection step, performed using 4 mL of deprotection cocktail with MW cycle 14. Cleavage was done with 10 mL of TFA/water/TIS (95:2.5:2.5) for 2 h. The resin was removed by filtration and the filtrate was concentrated by N_2 flow. The crude product was precipitated with cold diethyl ether, centrifuged (4,000 g for 10 min) and the supernatant was discarded. The precipitate was dissolved in 0.2 M phosphate buffer, containing 6 M GdmCl and the crude product was converted into the corresponding tipphenol thioester as previously described.[1] The desired product was then isolated by HPLC. Product was characterized at the LC/MS.

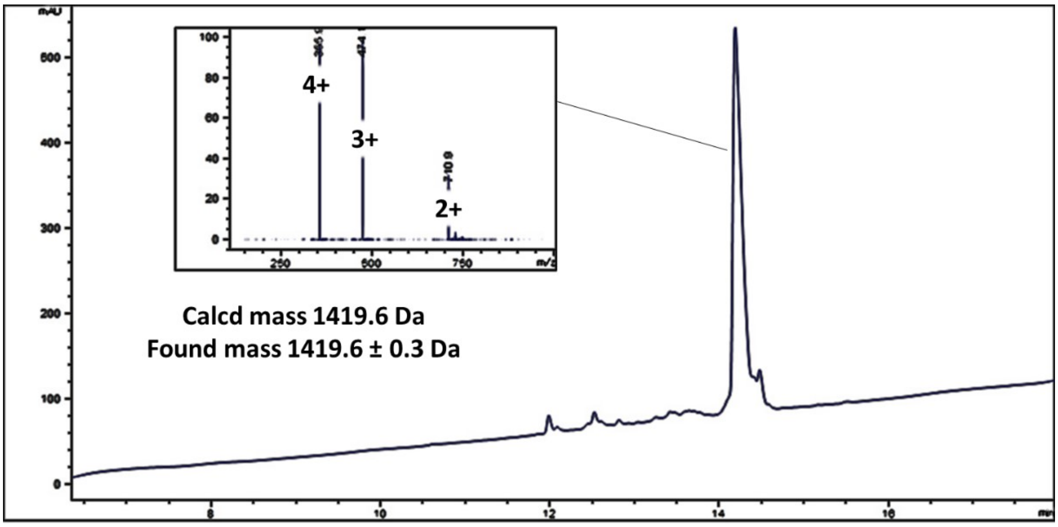

LC chromatogram recorded at 214 nm (+ mass spectrum of the indicated peak) of peptide **4c**. Column: *ACE 3 C18*, 2.1 x 100 mm, 3 µm particle size, 300 Å pore size; Method: flow rate = 0.5 mL · min^{-1}, H_2O: CH_3CN, 0.1% HCOOH, 95:5 for 2 min → 95:5 to 43:57 over 13 min → 5:95 for 7 min.

1.7 SPPS procedure for peptide 5a

Synthesis scale: 0.05 mmol. Resin: Rink Amide ProTide Resin (LL), loading 0.19 mmol/g – 263 mg. HATU concentration in DMF: 0.5 M. DIPEA concentration in NMP: 2 M. Deprotection cocktail: 20% (v/v) piperidine in DMF + 1% (v/v) formic acid. Fmoc-MeDbz-OH in DMF: 0.05 M. Fmoc-Asp(OtBu)-(Dmb)Gly-OH in DMF: 0.15 M. Amino acids in DMF: 0.33 M. Prior to the start of the synthesis, the resin is swollen in the reaction vessel of the automated synthesizer using 5 mL of DMF for 5 min at rt.

**Reagent**	**Method**	**Reagent concentration during coupling step (mM)**
Fmoc-Gly-OH	20	167
Fmoc-MeDbz-OH	14	35
Fmoc-Ala-OH	20	167
Fmoc-Gly-OH	21	167
Fmoc-Trp(Boc)-OH	20	167
Fmoc-His(Trt)-OH	20	167
Fmoc-His(Trt)-OH	19	167
Fmoc-Ser(tBu)-OH	18	167
Fmoc-Gly-OH	17	167
Fmoc-Gly-OH	17	167
Fmoc-Arg(Pbf)-OH	16	167
Fmoc-Leu-OH	16	167
Fmoc-Arg(Pbf)-OH	16	167
Fmoc-Leu-OH	16	167
Fmoc-Val-OH	16	167
Fmoc-Leu-OH	16	167
Fmoc-His(Trt)-OH	16	167
Fmoc-Leu-OH	16	167
Fmoc-Thr(tBu)-OH	8	167
Fmoc-Ser(tBu)-OH	8	167
Fmoc-Glu(OtBu)-OH	8	167
Fmoc-Lys(Boc)-OH	8	167
Fmoc-Gln(Trt)-OH	8	167
Fmoc-Ile-OH	8	167

Fmoc-Asn(Trt)-OH	8	167
Fmoc-Tyr(tBu)-OH	8	167
Fmoc-Asp(OtBu)-OH	8	167
Fmoc-Ser(tBu)-OH	8	167
Fmoc-Leu-OH	8	167
Fmoc-Thr(tBu)-OH	8	167
Fmoc-Arg(Pbf)-OH	8	167
Fmoc-Asp(OtBu)-(Dmb)Gly-OH	15	107
Fmoc-Glu(OtBu)-OH	8	167
Fmoc-Leu-OH	8	167
Fmoc-Gln(Trt)-OH	8	167
Fmoc-Lys(Boc)-OH	8	167
Fmoc-Gly-OH	8	167
Boc-Thz-OH	8	167

After the last coupling, the MeDbz group is activated into MeNbz by incubating the resin with 5 equivalents of 4-nitrophenyl chloroformate in DCM (3 x 30 min), followed by 0.5 M DIPEA in DMF (3 x 10 min). Cleavage was done with 10 mL of TFA/water/TIS (95:2.5:2.5) for 2 h. The resin was removed by filtration and the filtrate was concentrated by N_2 flow. The crude product was precipitated with cold diethyl ether, centrifuged (4,000 g for 10 min) and the supernatant was discarded. The precipitate was dissolved in water/acetonitrile (1:1) + 0.1% TFA and purified by HPLC. Product was characterized at the LC/MS.

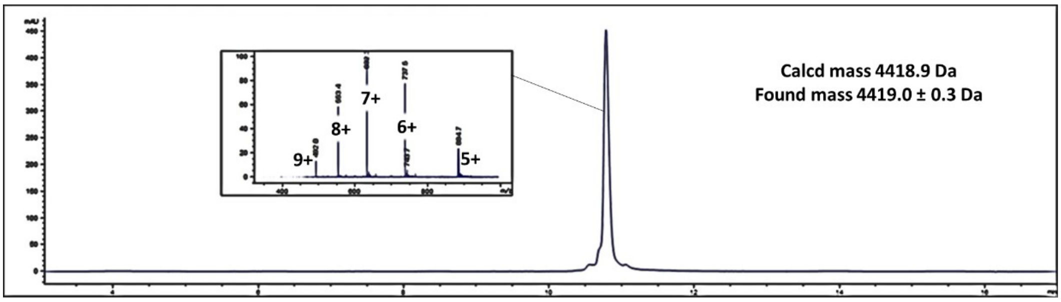

LC chromatogram recorded at 214 nm (+ mass spectrum of the indicated peak) of peptide **5a**. Column: *ACE 3 C18*, 2.1 x 100 mm, 3 µm particle size, 300 Å pore size; Method: flow rate = 0.5 mL · min^{-1}, H_2O: CH_3CN, 0.1% HCOOH, 95:5 for 2 min → 95:5 to 55:45 over 10 min → 5:95 for 5 min.

1.8 SPPS procedure for peptide 5b

Synthesis scale: 0.05 mmol. Resin: Rink Amide ProTide Resin (LL), loading 0.19 mmol/g – 263 mg. DIC concentration in DMF: 1 M. HOBt concentration in DMF: 1 M. Deprotection cocktail: 20% (v/v) piperidine in DMF + 1% (v/v) formic acid. Fmoc-MeDbz-OH in DMF: 0.05 M. Amino acids in DMF: 0.33 M. Prior to the start of the synthesis, the resin is swollen in the reaction vessel of the automated synthesizer using 5 mL of DMF for 5 min at rt.

**Reagent**	**Method**	**Reagent concentration during coupling step (mM)**
Fmoc-Gly-OH	22	167
Fmoc-MeDbz-OH	22	35
Fmoc-Phe-OH	22	167
Fmoc-Ile-OH	22	167
Fmoc-Leu-OH	22	167
Fmoc-Arg(Pbf)-OH	22	167
Fmoc-Gln(Trt)-OH	22	167
Fmoc-Gln(Trt)-OH	22	167

Fmoc-Asp(OtBu)-OH	22	167
Fmoc-Pro-OH	23	167
Fmoc-Pro-OH	23	167
Fmoc-Ile-OH	24	167
Fmoc-Gly-OH	24	167
Fmoc-Glu(OtBu)-OH	24	167
Fmoc-Lys(Boc)-OH	24	167
Fmoc-Asp(OtBu)-OH	24	167
Fmoc-Gln(Trt)-OH	24	167
Fmoc-Ile-OH	24	167
Fmoc-Lys(Boc)-OH	24	167
Boc-Thz-OH	24	167

After the last coupling, the MeDbz group is activated into MeNbz by incubating the resin with 5 equivalents of 4-nitrophenyl chloroformate in DCM (3 x 30 min), followed by 0.5 M DIPEA in DMF (3 x 10 min). Cleavage was done with 10 mL of TFA/water/TIS (95:2.5:2.5) for 2 h. The resin was removed by filtration and the filtrate was concentrated by N_2 flow. The crude product was precipitated with cold diethyl ether, centrifuged (4,000 g for 10 min) and the supernatant was discarded. The precipitate was dissolved in water/acetonitrile (1:1) + 0.1% TFA and purified by HPLC. Product was characterized at the LC/MS.

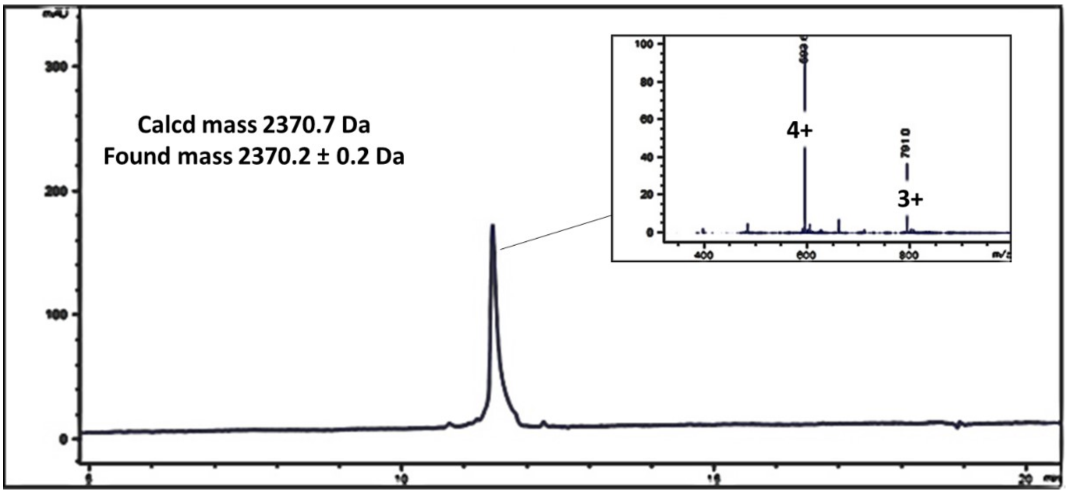

LC chromatogram recorded at 214 nm (+ mass spectrum of the indicated peak) of peptide **5b**. Column: *ACE 3 C18*, 2.1 x 100 mm, 3 µm particle size, 300 Å pore size; Method: flow rate = 0.5 mL · min^{-1}, H_2O: CH_3CN, 0.1% HCOOH, 95:5 for 2 min → 95:5 to 43:57 over 13 min → 5:95 for 7 min.

1.9 SPPS procedure for peptide 5c

Synthesis scale: 0.05 mmol. Resin: Rink Amide ProTide Resin (LL), loading 0.19 mmol/g – 263 mg. HATU concentration in DMF: 0.5 M. DIPEA concentration in NMP: 2 M. Deprotection cocktail: 20% (v/v) piperidine in DMF + 1% (v/v) formic acid. Fmoc-MeDbz-OH in DMF: 0.05 M. Fmoc-amino acids in DMF: 0.33 M. Prior to the start of the synthesis, the resin is swollen in the reaction vessel of the automated synthesizer using 5 mL of DMF for 5 min at rt.

**Reagent**	**Method**	**Reagent concentration during coupling step (mM)**
Fmoc-Gly-OH	12	167
Fmoc-MeDbz-OH	14	35
Fmoc-Lys(Boc)-OH	12	167
Fmoc-Val-OH	12	167
Fmoc-Asn(Trt)-OH	12	167
Fmoc-Glu(OtBu)-OH	12	167

Fmoc-Ile-OH	12	167
Fmoc-Thr(tBu)-OH	13	167
Fmoc-Asp(OtBu)-OH	12	167
Fmoc-Ser(tBu)-OH	8	167
Fmoc-Pro-OH	11	167
Fmoc-Glu(OtBu)-OH	8	167
Fmoc-Val-OH	8	167
Fmoc-Glu(OtBu)-OH	8	167
Fmoc-Leu-OH	8	167
Fmoc-Thr(tBu)-OH	10	167
Fmoc-Ile-OH	9	167
Fmoc-Thr(tBu)-OH	10	167
Fmoc-Lys(Boc)-OH	8	167
Fmoc-Gly-OH	8	167
Fmoc-Thr(tBu)-OH	10	167
Fmoc-Leu-OH	8	167
Fmoc-Thr(tBu)-OH	10	167
Fmoc-Lys(Boc)-OH	8	167
Fmoc-Val-OH	9	167
Fmoc-Phe-OH	8	167
Fmoc-Ile-OH	9	167
Fmoc-Gln(Trt)-OH	8	167
Boc-NLe-OH	8	167

After the last coupling, the MeDbz group is activated into MeNbz by incubating the resin with 5 equivalents of 4-nitrophenyl chloroformate in DCM (3 x 30 min), followed by 0.5 M DIPEA in DMF (3 x 10 min). Cleavage was done with 10 mL of TFA/water/TIS (95:2.5:2.5) for 2 h. The resin was removed by filtration and the filtrate was concentrated by N_2 flow. The crude product was precipitated with cold diethyl ether, centrifuged (4,000 g for 10 min) and the supernatant was discarded. The precipitate was dissolved in water/acetonitrile (1:1) + 0.1% TFA and purified by HPLC. Product was characterized at the LC/MS.

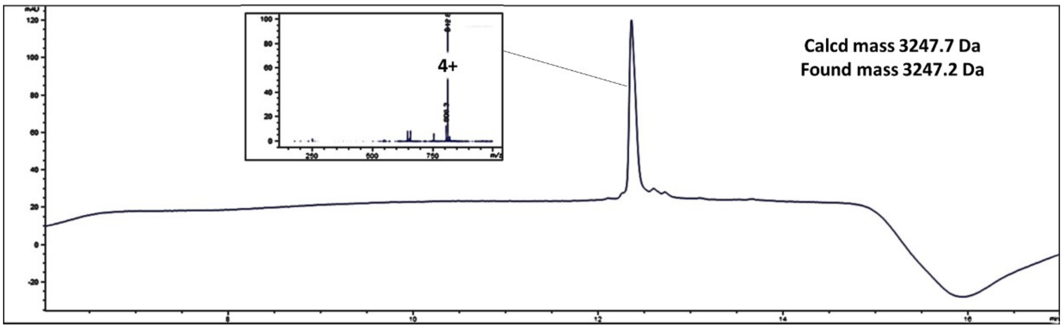

LC chromatogram recorded at 214 nm (+ mass spectrum of the indicated peak) of peptide **5c**. Column: *ACE 3 C18*, 2.1 x 100 mm, 3 µm particle size, 300 Å pore size; Method: flow rate = 0.5 $mL \cdot min^{-1}$, H_2O: CH_3CN, 0.1% HCOOH, 95:5 for 2 min → 95:5 to 55:45 over 10 min → 5:95 for 5 min.

2. LC/MS chromatograms for the synthesis of compounds **4**

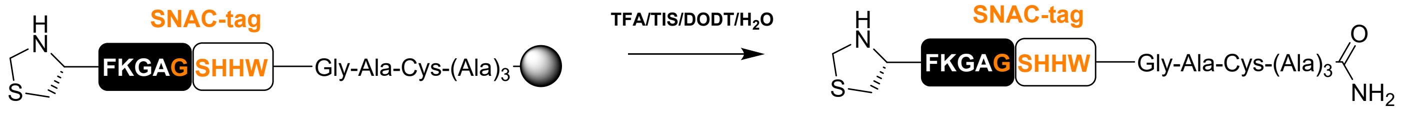

calculated molecular weight: 1584.8 Da

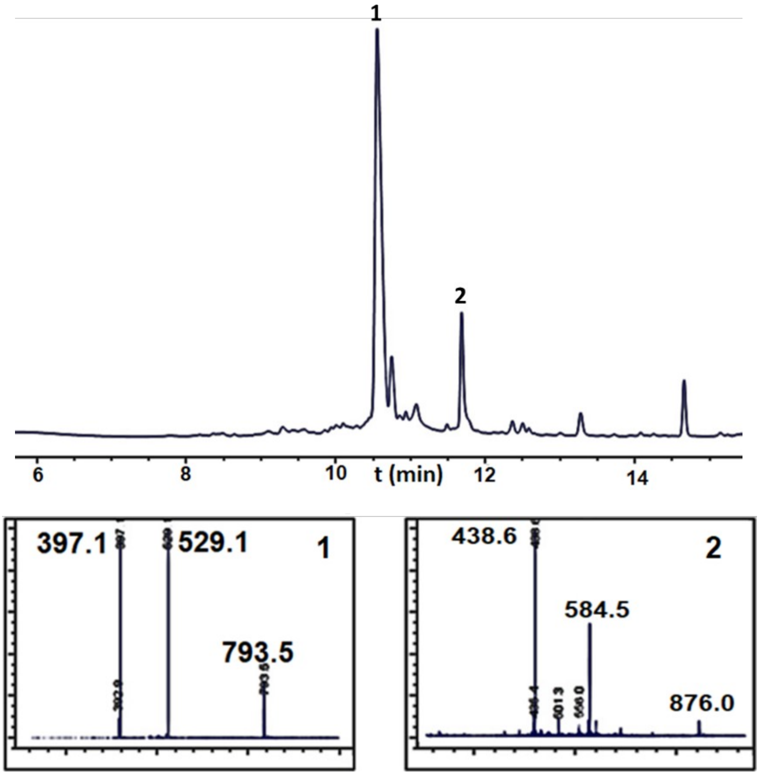

peak #	found mass	peak assignment
1	1584.6 ± 0.3	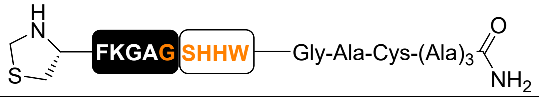
2	1750.3 ± 0.2	?

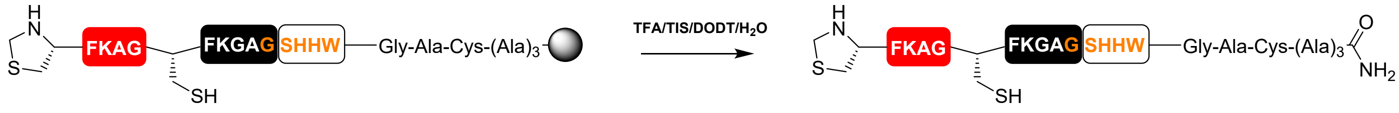

calculated molecular weight: 2091.4 Da

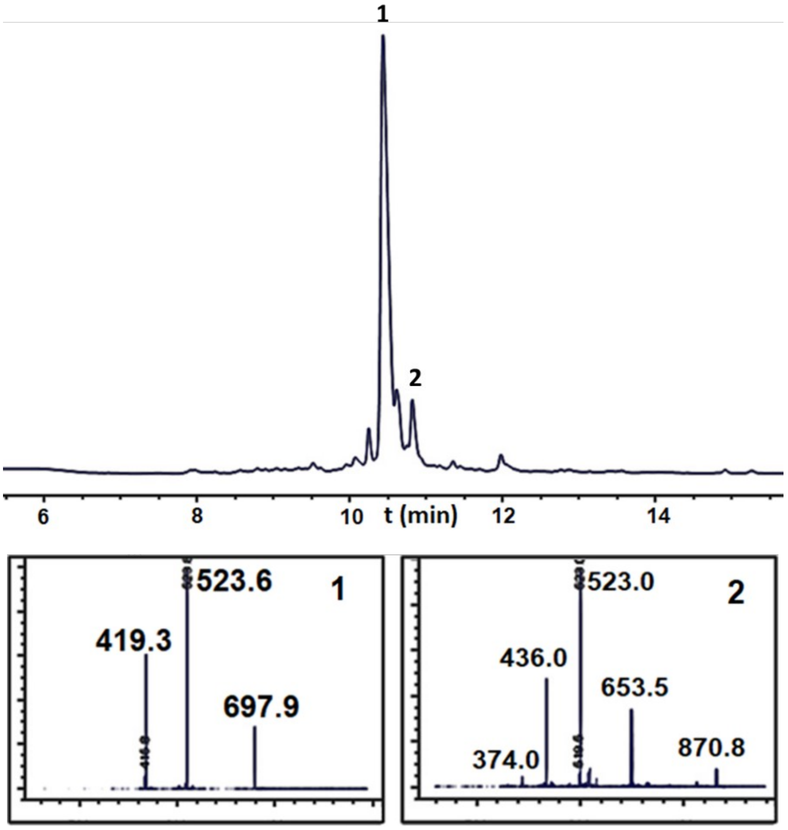

peak #	found mass	peak assignment
1	2090.9 ± 0.4	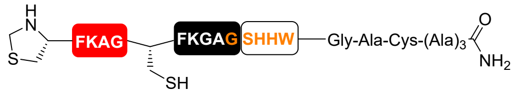
2	2610.1 ± 0.4	?

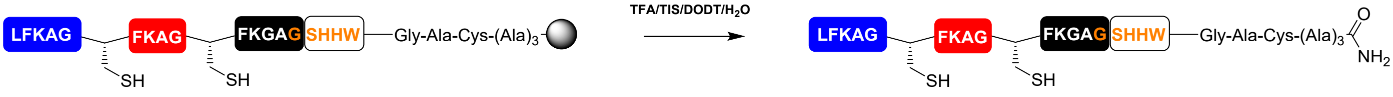

calculated molecular weight: 2596.0 Da

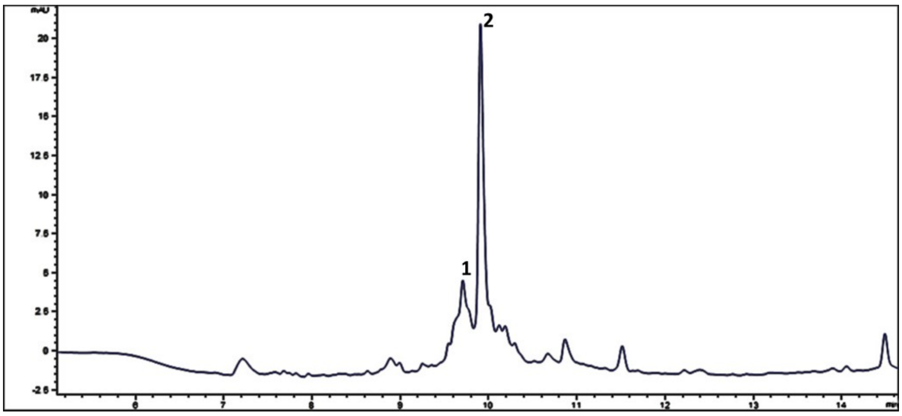

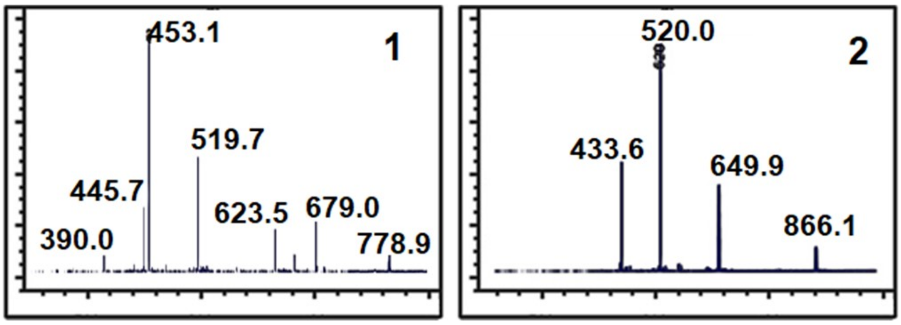

peak #	found mass	peak assignment
1	3112.3 ± 0.3	?
2	2595.4 ± 0.2	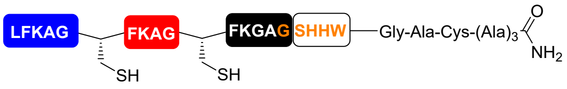

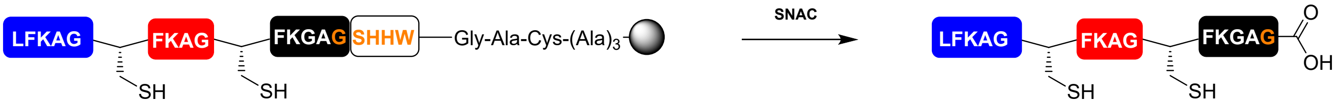

calculated molecular weight: 1605.0 Da

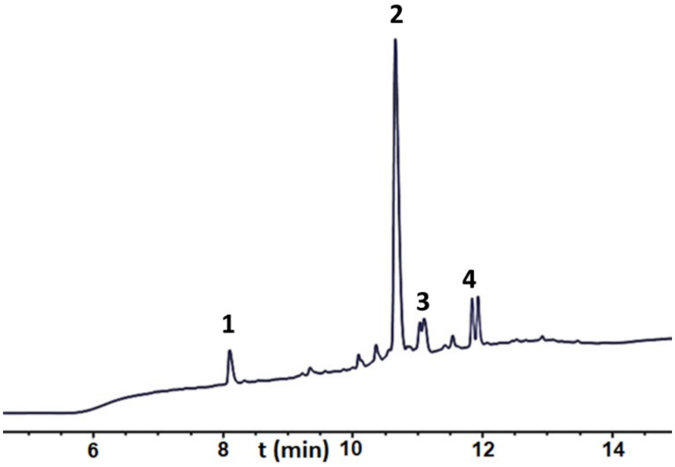

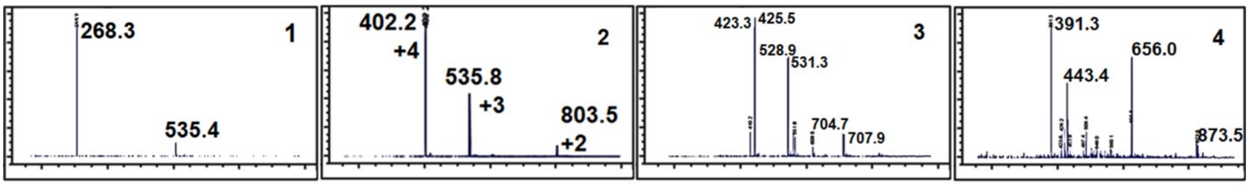

peak #	found mass	peak assignment
1	534.5 ± 0.1	?
2	1604.7 ± 0.2	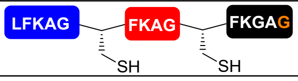
3	2111.4 ± 0.2 and 2121.8 ± 0.8	?
4	?	?

3. LC/MS chromatograms for the synthesis of compounds **5**

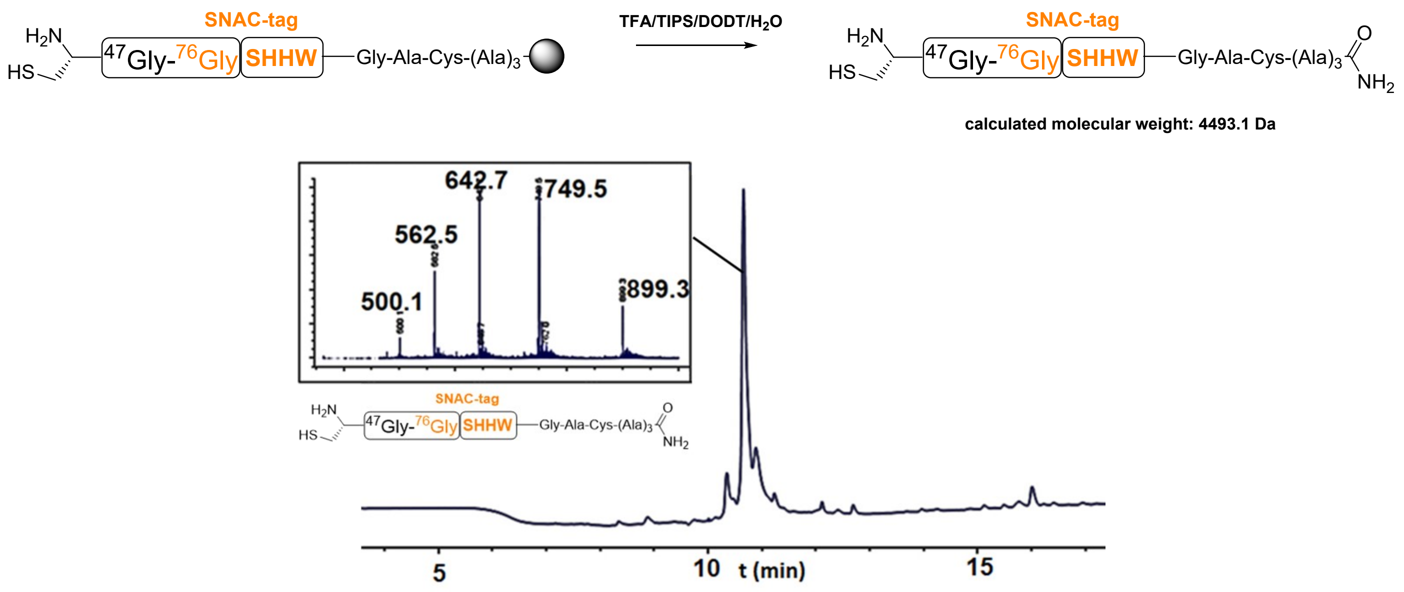

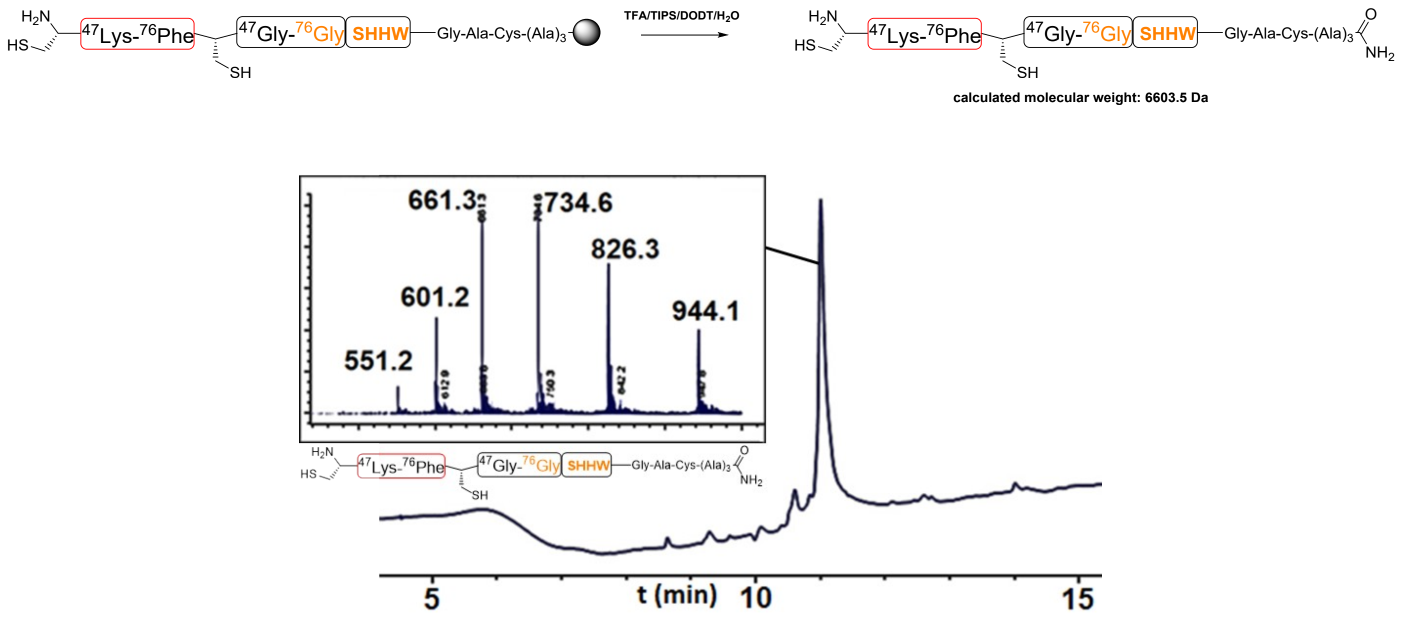

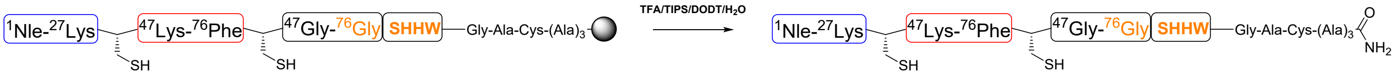

calculated molecular weight: 9603.0 Da

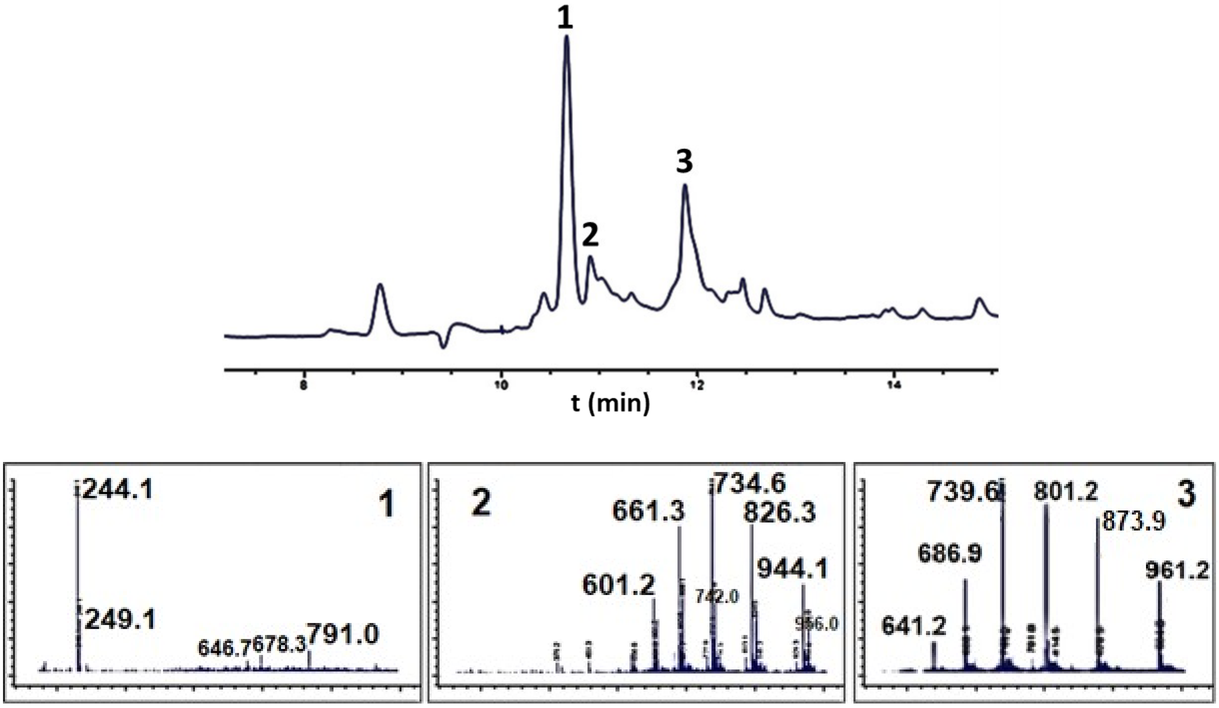

peak #	found mass	peak assignment
1	?	?
2	6602.3 ± 0.4	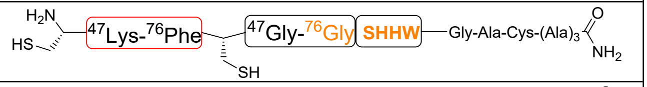
3	9602.3 ± 0.4	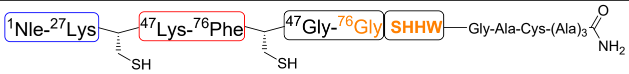

4. Supplementary figures

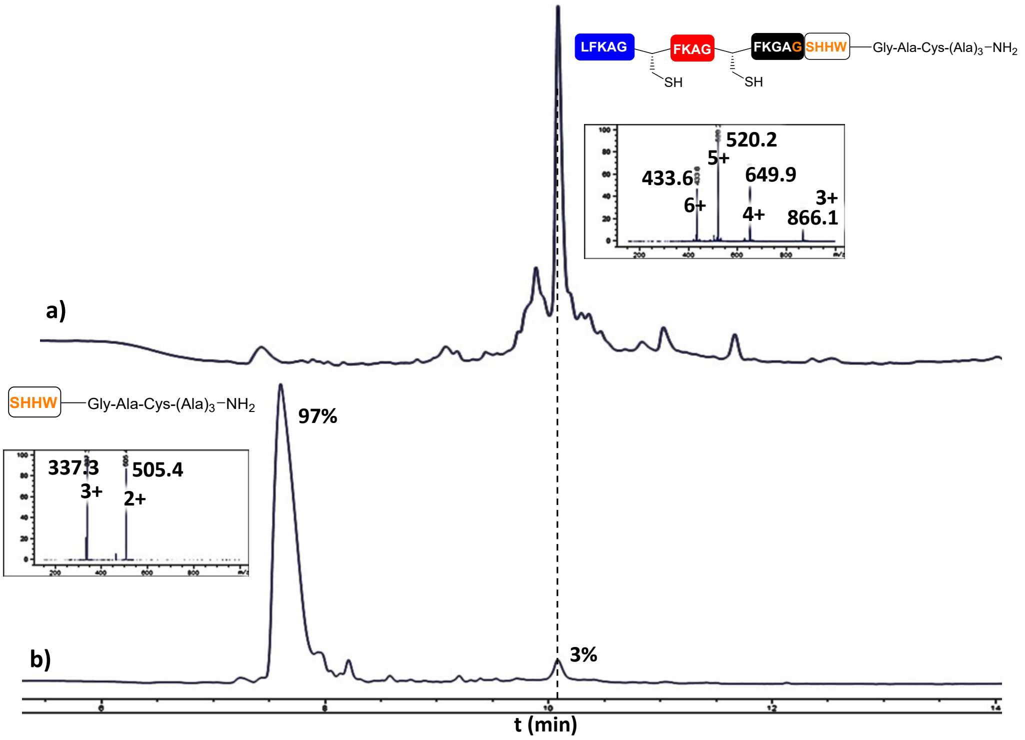

Supplementary figure 1. LC chromatogram recorded at 280 nm of TFA cleavages of polypeptide **4** before (a) and after (b) SNAC. Percentage of cleavage is worked out by integrating the areas of the peak corresponding to the cleaved linker and to the peptide remained on the resin. Column: *ACE 3 C18*, 2.1 x 100 mm, 3 µm particle size, 300 Å pore size; Method: flow rate = 0.5 mL · min^{-1}, H_2O: CH_3CN, 0.1% HCOOH, 95:5 for 2 min → 95:5 to 43:57 over 13 min → 5:95 for 7 min.

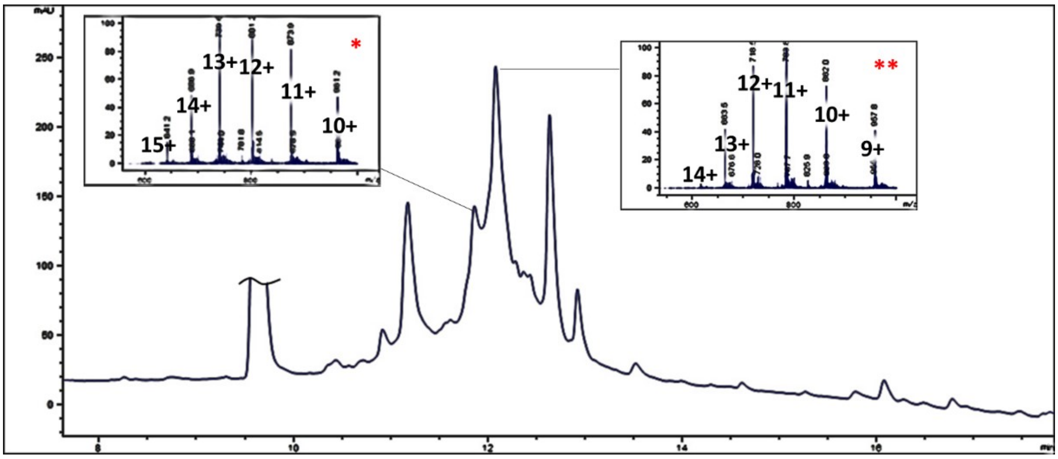

Supplementary figure 2. LC chromatogram recorded at 214 nm of TFA cleavages after SNAC for the synthesis of compound **5**. * denotes uncleaved intermediate **5c***, ** denotes cleaved product **5**.

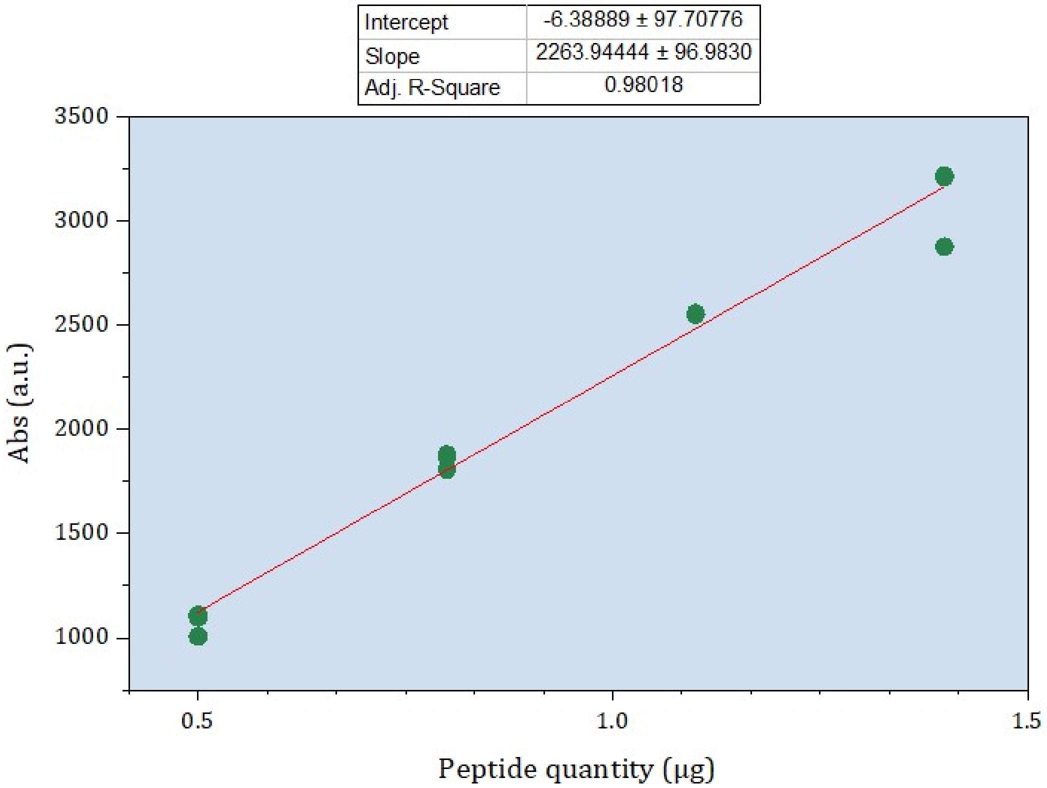

Supplementary figure 3. Calibration curve for polypeptide H-LFKAGCFKAGCFKGAG-NH_2. The plot was obtained by plotting the absorbance obtained at the LC/MS of a stock solution of H-LFKAGCFKAGCFKGAG-NH_2. For each quantity point, LC/MS run was repeated three times. The calibration curve was obtained by fitting the data points using Origin, version 2019b, OriginLab Corporation, Northampton, MA, USA.

5. References

1. J.-S. Zheng, S. Tang, Y.-K. Qi, Z.-P. Wang and L. Liu, *Nat. Protoc.*, 2013, **8**, 2483-2495.
2. D. T. Flood, J. C. J. Hintzen, M. J. Bird, P. A. Cistrone, J. S. Chen and P. E. Dawson, *Angew. Chem. Int. Ed.*, 2018, **57**, 11634-11639.
